# Supplementary material for: “Bringing greater research fluency into our educational vision”: A qualitative research study on improving Traditional Chinese Medicine research education
Source: PLoS One. 2024 Dec 19;19(12):e0312083. doi: 10.1371/journal.pone.0312083 (PMC11658634; doi:10.1371/journal.pone.0312083)
Supplement: S1 File — (DOCX) [file pone.0312083.s001.docx]

**Research Papers Recommended by SMEs**

1. **World View: different ways of knowing**

Cassidy, C. (2009). Model fit validity: seeking a balanced equation. The Journal of alternative and complementary medicine, 15(12), 1265-1266.

Cassidy, C. M. (2001). Beyond numbers: Qualitative research methods for Oriental medicine. Clinical Acupuncture: Scientific Basis, 151-169.

Cassidy, C.M.   Chinese Medicine Users in the United States:

                     Part I, Utilization, Satisfaction, Medical Plurality. *J Altern Comple Med 4(1):17-28,*

                     Part II: Preferred Aspects of Care.*J Altern Comple Med* 4(2):189-202, 1998.

Cassidy, C. (2010). How acupuncture is actually practiced, and why this matters to clinical research design. European Journal of Oriental Medicine, 6(4), 20-25.

MacPherson, H., Sinclair-Lian, N., & Thomas, K. (2006). Patients seeking care from acupuncture practitioners in the UK: a national survey. Complementary therapies in medicine, 14(1), 20-30.[or others with H. Macpherson].

Bell, I. R., Koithan, M., & Pincus, D. (2012). Methodological implications of nonlinear dynamical systems models for whole systems of complementary and alternative medicine. Forschende Komplementarmedizin (2006), 19 Suppl 1, 15–21. <https://doi.org/10.1159/000335183>

Schön, Donald A. (1983). The reflective practitioner: how professionals think in action. New York: Basic Books. ISBN 978-0465068746. OCLC 8709452.

Hendricks, Joyce; Mooney, Deborah; Berry, Catherine (April 1996). "A practical strategy approach to use of reflective practice in critical care nursing". Intensive and Critical Care Nursing. 12 (2): 97–101. doi:10.1016/S0964-3397(96)81042-1. PMID 8845631.

1. **Challenges in acupuncture research**

Langevin, H. M., Wayne, P. M., MacPherson, H., Schnyer, R., Milley, R. M., Napadow, V., ... & Hammerschlag, R. (2010). Paradoxes in acupuncture research: strategies for moving forward. Evidence-Based Complementary and Alternative Medicine, 2011.

Langevin, H. M., Schnyer, R., MacPherson, H., Davis, R., Harris, R. E., Napadow, V., ... & Executive Board of the Society for Acupuncture Research. (2015). Manual and electrical needle stimulation in acupuncture research: pitfalls and challenges of heterogeneity. The Journal of Alternative and Complementary Medicine, 21(3), 113-128.

Vickers, A. J., Vertosick, E. A., Lewith, G., MacPherson, H., Foster, N. E., Sherman, K. J., Irnich, D., Witt, C. M., Linde, K., & Acupuncture Trialists’ Collaboration (2021). Do the effects of acupuncture vary between acupuncturists? Analysis of the Acupuncture Trialists' Collaboration individual patient data meta-analysis. *Acupuncture in medicine : journal of the British Medical Acupuncture Society*, *39*(4), 309–317. https://doi.org/10.1177/0964528420959089

Kaptchuk. Placebo effects in acupuncture. Med Acupuncture 2020; 32: 352-355.

Kaptchuk, T. J., Kelley, J. M., Conboy, L. A., Davis, R. B., Kerr, C. E., Jacobson, E. E., ... & Lembo, A. J. (2008). Components of placebo effect: randomised controlled trial in patients with irritable bowel syndrome. Bmj, 336(7651), 999-1003.

Kaptchuk, T. J., Stason, W. B., Davis, R. B., Legedza, A. R., Schnyer, R. N., Kerr, C. E., ... & Goldman, R. H. (2006). Sham device v inert pill: randomised controlled trial of two placebo treatments. Bmj, 332(7538), 391-397.(Possibly just abstract.)

Wechsler, M. E., Kelley, J. M., Boyd, I. O. E., Dutile, S., Marigowda, G., Kirsch, I., & Israel, E. K. T. (2011). Active or placebo albuterol, sham acupuncture or no treatment in asthma. N Engl J Med, 365, 119-126. (Possibly just abstract.)

Taylor-Swanson, L., Prasad, T., & Conboy, L. (2019). Complex adaptive systems theory and inter-rater reliability: Proposed answers to challenging questions. The Journal of Alternative and Complementary Medicine, 25(11), 1074-1076.

Witt, C. M. (2009). Efficacy, effectiveness, pragmatic trials–guidance on terminology and the advantages of pragmatic trials. Complementary Medicine Research, 16(5), 292-294.

Greenhalgh, T., Howick, J., & Maskrey, N. (2014). Evidence based medicine: a movement in crisis?. Bmj, 348.

Pollitt, A., Ahmed, A., & Baird, J. A. (2008). Archived Content. (This document provides guidance on the development, evaluation and implementation of complex interventions to improve health.)

Jacobson E, Conboy L, Tsering D, Shields M, McKnight P, Wayne PM, Schnyer R. Experimental Studies of Inter-Rater Agreement in Traditional Chinese Medicine: A Systematic Review. J Altern Complement Med. 2019 Nov;25(11):1085-1096. doi: 10.1089/acm.2019.0197. PMID: 31730402; PMCID: PMC6864748.

1. **Clinical application of acupuncture:**
2. Whole Person Research: what we have learned from clinical trials on acupuncture’s effect on the sense of well-being of patients; qualitative research on patients’ perspectives.

Ritenbaugh, C., Hammerschlag, R., Calabrese, C., Mist, S., Aickin, M., Sutherland, E., ... & Dworkin, S. F. (2008). A pilot whole systems clinical trial of traditional Chinese medicine and naturopathic medicine for the treatment of temporomandibular disorders. The Journal of Alternative and Complementary Medicine, 14(5), 475-487.

Kligler, B., Buonora, M., Gabison, J., Jacobs, E., Karasz, A., & McKee, M. D. (2015). “I felt like it was god's hands putting the needles in”: A qualitative analysis of the experience of acupuncture for chronic pain in a low-income, ethnically diverse, and medically underserved patient population. The Journal of Alternative and Complementary Medicine, 21(11), 713-719.

Paterson, C., & Britten, N. (2004). Acupuncture as a complex intervention: a holistic model. Journal of Alternative & Complementary Medicine, 10(5), 791-801

Stibich, M., & Wissow, L. (2006). Meaning shift: findings from wellness acupuncture. Alternative therapies in health and medicine, 12(2), 42.

Taylor-Swanson, L., Altschuler, D., Taromina, K., Anderson, B., Bensky, D., Cohen, M., ... & Conboy, L. (2022). SEAttle-based research of Chinese herbs for COVID-19 study: A whole health perspective on Chinese herbal medicine for symptoms that may be related to COVID-19. *Global Advances in Health and Medicine*, *11*, 21649561211070483.

Langevin, H. M. (2022). Making connections to improve health outcomes. Global advances in health and medicine, 11, 2164957X221079792.

1. Pain

Vickers, A. J., Vertosick, E. A., Lewith, G., MacPherson, H., Foster, N. E., Sherman, K. J., Irnich, D., Witt, C. M., Linde, K., & Acupuncture Trialists' Collaboration (2018). Acupuncture for Chronic Pain: Update of an Individual Patient Data Meta-Analysis. The journal of pain, 19(5), 455–474. https://doi.org/10.1016/j.jpain.2017.11.005

Cummings, M. (2009). Modellvorhaben Akupunktur–a summary of the ART, ARC and GERAC trials. Acupuncture in Medicine, 27(1), 26-30. (In October 2000 the German Federal Committee of Physicians and Health Insurers recommended that special Model Projects on Acupuncture (‘‘Modellvorhaben Akupunktur’’) be developed in order to determine the evidence-based role of acupuncture in the treatment of certain illnesses. This paper presents a summary of the main randomised controlled trials performed as part of these projects, and the associated economic analyses.)

Weidenhammer, W., Streng, A., Linde, K., Hoppe, A., & Melchart, D. (2007). Acupuncture for chronic pain within the research program of 10 German Health Insurance Funds--basic results from an observational study. Complementary therapies in medicine, 15(4), 238–246. https://doi.org/10.1016/j.ctim.2006.09.005

Molsberger, A. F., Schneider, T., Gotthardt, H., & Drabik, A. (2010). German Randomized Acupuncture Trial for chronic shoulder pain (GRASP)–a pragmatic, controlled, patient-blinded, multi-centre trial in an outpatient care environment. Pain, 151(1), 146-154.

Shin, J. S., Ha, I. H., Lee, J., Choi, Y., Kim, M. R., Park, B. Y., Shin, B. C., & Lee, M. S. (2013). Effects of motion style acupuncture treatment in acute low back pain patients with severe disability: a multicenter, randomized, controlled, comparative effectiveness trial. Pain, 154(7), 1030–1037. <https://doi.org/10.1016/j.pain.2013.03.013>

Conboy L, Gerke T, Hsu KY, St John M, Goldstein M, Schnyer R. The Effectiveness of Individualized Acupuncture Protocols in the Treatment of Gulf War Illness: A Pragmatic Randomized Clinical Trial. PLoS One. 2016 Mar 31;11(3):e0149161. doi: 10.1371/journal.pone.0149161. PMID: 27031099; PMCID: PMC4816551.

Osteo-Arthritis of the Knee

Brian Berman, Lixing Lao, Patricia Langenberg, Wen Lin Lee , Adele M.K. Gilpin, Marc C. Hochberg. Effectiveness of Acupuncture as Adjunctive Therapy in Osteoarthritis of the Knee: A Randomized, Controlled Trial. Ann Intern Med 2004;141:901-10.

Berman, B. M., Singh, B. B., Lao, L., Langenberg, P., Li, H., Hadhazy, V., ... & Hochberg, M. (1999). A randomized trial of acupuncture as an adjunctive therapy in osteoarthritis of the knee. Rheumatology (Oxford, England), 38(4), 346-354.

Manheimer, E., Linde, K., Lao, L., Bouter, L. M., & Berman, B. M. (2007). Meta-analysis: acupuncture for osteoarthritis of the knee. Annals of internal medicine, 146(12), 868-877.

Cancer-related pain

Hershman, D. L., Unger, J. M., Greenlee, H., Capodice, J. L., Lew, D. L., Darke, A. K., ... & Crew, K. D. (2018). Effect of acupuncture vs sham acupuncture or waitlist control on joint pain related to aromatase inhibitors among women with early-stage breast cancer: a randomized clinical trial. Jama, 320(2), 167-176.

Mao, J. J., Liou, K. T., Baser, R. E., Bao, T., Panageas, K. S., Romero, S. A., ... & Kantoff, P. W. (2021). Effectiveness of electroacupuncture or auricular acupuncture vs usual care for chronic musculoskeletal pain among cancer survivors: the PEACE randomized clinical trial. JAMA oncology, 7(5), 720-727.

He, Y., Guo, X., May, B. H., Zhang, A. L., Liu, Y., Lu, C., ... & Zhang, H. (2020). Clinical evidence for association of acupuncture and acupressure with improved cancer pain: a systematic review and meta-analysis. JAMA oncology, 6(2), 271-278.

Non-pain condition: Hot Flashes: Lesi, Grazia; Razzini, Giorgia; Musti, Muriel Assunta, et al. Acupuncture As an Integrative Approach for the Treatment of Hot Flashes in Women With Breast Cancer: A Prospective Multicenter Randomized Controlled Trial (AcCliMaT). J Clin Oncol. 2016 May 20;34(15):1795-802.

1. High Blood Pressure

Longhurst, J. C., & Tjen-A-Looi, S. C. (2017). Evidence-based blood pressure reducing actions of electroacupuncture: mechanisms and clinical application. *Sheng li xue bao : [Acta physiologica Sinica]*, *69*(5), 587–597. ( This review summarizes the series of studies on acupuncture-inhibition of increased sympathetic activity. Based on neuronal processing, neuronal pathways and circuitry, specific neurotransmitter systems, and stimulation parameters and sites, the targeted acupuncture treatment decreased blood pressure in hypertensive subjects.)

Li, P., Tjen-A-Looi, S. C., Cheng, L., Liu, D., Painovich, J., Vinjamury, S., & Longhurst, J. C. (2015). Long-Lasting Reduction of Blood Pressure by Electroacupuncture in Patients with Hypertension: Randomized Controlled Trial. Medical acupuncture, 27(4), 253–266. <https://doi.org/10.1089/acu.2015.1106> (The study tested the findings, observed in animal studies, in hypertensive patients not on anti-hypertensive medication.)

1. Integrative Oncology and Cancer Symptoms:

Witt, C. M., Balneaves, L. G., Cardoso, M. J., Cohen, L., Greenlee, H., Johnstone, P., ... & Mao, J. J. (2017). A comprehensive definition for integrative oncology. JNCI monographs, 2017(52).

Zia, F. Z., Olaku, O., Bao, T., Berger, A., Deng, G., Yin Fan, A., ... & Mao, J. J. (2017). The National Cancer Institute’s conference on acupuncture for symptom management in oncology: state of the science, evidence, and research gaps. JNCI Monographs, 2017(52).

Molassiotis, A., Bardy, J., Finnegan-John, J., Mackereth, P., Ryder, D. W., Filshie, J., ... & Richardson, A. (2012). Acupuncture for cancer-related fatigue in patients with breast cancer: a pragmatic randomized controlled trial. Journal of Clinical Oncology, 30(36), 4470-4476.

Garland, S. N., Xie, S. X., DuHamel, K., Bao, T., Li, Q., Barg, F. K., ... & Mao, J. J. (2019). Acupuncture versus cognitive behavioral therapy for insomnia in cancer survivors: a randomized clinical trial. JNCI: Journal of the National Cancer Institute, 111(12), 1323-1331.

Mao, J. J., Bowman, M. A., Xie, S. X., Bruner, D., DeMichele, A., & Farrar, J. T. (2015). Electroacupuncture versus gabapentin for hot flashes among breast cancer survivors: a randomized placebo-controlled trial. Journal of Clinical Oncology, 33(31), 3615.

Shen, J., Wenger, N., Glaspy, J., Hays, R. D., Albert, P. S., Choi, C., & Shekelle, P. G. (2000). Electroacupuncture for control of myeloablative chemotherapy–induced emesis: a randomized controlled trial. Jama, 284(21), 2755-2761.

Lesi, Grazia; Razzini, Giorgia; Musti, Muriel Assunta, et al. Acupuncture As an Integrative Approach for the Treatment of Hot Flashes in Women With Breast Cancer: A Prospective Multicenter Randomized Controlled Trial (AcCliMaT). J Clin Oncol. 2016 May 20;34(15):1795-802.

1. Acupuncture to improve the outcomes of IVF and during pregnancy:

Hullender Rubin LE, Anderson BJ, Craig LB. Acupuncture and in vitro fertilization research: current and future directions. Acupunct Med. 2018 Apr;36(2):117-122.

Hullender Rubin LE. Point of influence: What is the role of acupuncture and in vitro fertilization outcomes? Med Acupunct. 2019 Dec 1;31(6):329-333.

Hullender Rubin LE, Opsahl MS, Wiemer KE, Mist SD, Caughey AB. Impact of whole systems traditional Chinese medicine on in-vitro fertilization outcomes. Reprod Biomed Online. 2015 Jun;30(6):602-12.

Niemtzow, R. C., Betts, D., Budd, S., Citkovitz, C., Kocher, Z., & Mummery, C. (2019). Acupuncture During Pregnancy: An Expert Discussion. Medical acupuncture, 31(5), 251–258. https://doi.org/10.1089/acu.2019.29112.rtl

1. Acupuncture for Anxiety and Depression

Zhang, Z.-J., Chen, H.-Y., Yip, K.-C., Ng, R. & Wong, V. T. The effectiveness and

safety of acupuncture therapy in depressive disorders: Systematic review and meta-analysis. Journal of Affective Disorders 124, 9–21 (2010).

Smith, C. A., Armour, M., Lee, M. S., Wang, L. Q., & Hay, P. J. (2018). Acupuncture for depression. The Cochrane database of systematic reviews, 3(3), CD004046. <https://doi.org/10.1002/14651858.CD004046.pub4>

Tu, C. H., MacDonald, I., & Chen, Y. H. (2019). The Effects of Acupuncture on Glutamatergic Neurotransmission in Depression, Anxiety, Schizophrenia, and Alzheimer's Disease: A Review of the Literature. Frontiers in psychiatry, 10, 14. <https://doi.org/10.3389/fpsyt.2019.00014>

Schnyer, R Commentary of Cochrane ReviewFront. Pharmacol., 07 May 2020 | <https://cam.cochrane.org/sites/cam.cochrane.org/files/uploads/Schnyer%20Commentary%20Cochrane%20Review%20Acupuncture%20for%20Depression%202011.pdf>

Li, C., Huang, J., Cheng, Y. C., & Zhang, Y. W. (2020). Traditional Chinese Medicine in Depression Treatment: From Molecules to Systems. Frontiers in pharmacology, 11, 586. <https://doi.org/10.3389/fphar.2020.00586>

MacPherson, H., Richmond, S., Bland, M., Brealey, S., Gabe, R., Hopton, A., Keding, A., Lansdown, H., Perren, S., Sculpher, M., Spackman, E., Torgerson, D., & Watt, I. (2013). Acupuncture and counselling for depression in primary care: a randomised controlled trial. PLoS medicine, 10(9), e1001518. <https://doi.org/10.1371/journal.pmed.1001518>

Pilkington K. (2010). Anxiety, depression and acupuncture: A review of the clinical research. Autonomic neuroscience : basic & clinical, 157(1-2), 91–95. <https://doi.org/10.1016/j.autneu.2010.04.002>

Hu, G. T., & Wang, Y. (2021). Advances in Treatment of Post-Traumatic Stress Disorder with Chinese Medicine. Chinese journal of integrative medicine, 27(11), 874–880. <https://doi.org/10.1007/s11655-021-2864-1>

Tu, C. H., MacDonald, I., & Chen, Y. H. (2019). The Effects of Acupuncture on Glutamatergic Neurotransmission in Depression, Anxiety, Schizophrenia, and Alzheimer's Disease: A Review of the Literature. Frontiers in psychiatry, 10, 14. <https://doi.org/10.3389/fpsyt.2019.00014>

Li, C., Huang, B., & Zhang, Y. W. (2021). Chinese Herbal Medicine for the Treatment of Depression: Effects on the Neuroendocrine-Immune Network. Pharmaceuticals (Basel, Switzerland), 14(1), 65. <https://doi.org/10.3390/ph14010065>

Khan, A., Faucett, J., Lichtenberg, P., Kirsch, I., & Brown, W. A. (2012). A systematic review of comparative efficacy of treatments and controls for depression. PloS one, 7(7), e41778. <https://doi.org/10.1371/journal.pone.0041778>

Duncan, A. D., & Kain, K. L. (2019). *The Tao of Trauma: A Practitioner's Guide for Integrating Five Element Theory and Trauma Treatment*. North Atlantic Books.

6. Battlefield acupuncture

Niemtzow, R. C. (2007). Battlefield acupuncture. Medical Acupuncture, 19(4), 225-228. <https://doi.org/10.1089/acu.2007.0603> https://www.liebertpub.com/doi/10.1089/acu.2007.0603

Niemtzow, R. C. (2007). Battlefield acupuncture. *Medical Acupuncture*, *19*(4), 225-228.

Castañeda, G., Romero, S., Mudra, S., Gingrich, T., & Levy, C. (2021). Provider Perceptions of Battlefield Acupuncture in a Major Veterans Health Administration Facility. *Medical Acupuncture*, *33*(2), 159-168

Niemtzow, R. C. (2020). Implementing battlefield acupuncture through a large medical system: overcoming barriers. Medical Acupuncture, 32(6), 377-380.

Jan, A. L., Aldridge, E. S., Rogers, I. R., Visser, E. J., Bulsara, M. K., & Niemtzow, R. C. (2017). Does ear acupuncture have a role for pain relief in the emergency setting? A systematic review and meta-analysis. Medical Acupuncture, 29(5), 276-289.

**IV. Physiology of acupuncture/Basic Science Research**

1. Nervous/Endocrine Systems/fMRI Studies

Zhang R, Lao L, Ren K, Berman BM. Mechanisms of Acupuncture–Electroacupuncture on Persistent Pain. Anesthesiology. 2014.120:482-503.

Huang W, Pach D, Napadow V, Park K, Long X, Neumann J, Maeda Y, Nierhaus T, Liang F, Witt CM. Characterizing acupuncture stimuli using brain imaging with FMRI--a systematic review and meta-analysis of the literature. PLoS One. 2012;7(4):e32960. doi: 10.1371/journal.pone.0032960. Epub 2012 Apr 9. PMID: 22496739; PMCID: PMC3322129.

Hui, K. K., Liu, J., Makris, N., Gollub, R. L., Chen, A. J., I. Moore, C., ... & Kwong, K. K. (2000). Acupuncture modulates the limbic system and subcortical gray structures of the human brain: evidence from fMRI studies in normal subjects. Human brain mapping, 9(1), 13-25.

Maeda, Y., Kim, H., Kettner, N., Kim, J., Cina, S., Malatesta, C., ... & Napadow, V. (2017). Rewiring the primary somatosensory cortex in carpal tunnel syndrome with acupuncture. Brain, 140(4), 914-927.

Chiang, C. Y. (1973). Peripheral afferent pathway for acupuncture analgesia. Sci. Sin., 16, 210-217.

Guo, Z. L., Fu, L. W., Su, H. F., Tjen-A-Looi, S. C., & Longhurst, J. C. (2018). Role of TRPV1 in acupuncture modulation of reflex excitatory cardiovascular responses. American journal of physiology. Regulatory, integrative and comparative physiology, 314(5), R655–R666. <https://doi.org/10.1152/ajpregu.00405.2017> (A mechanistic study on the local actions at the acupoint during manual acupuncture and electroacupuncture.)

Citkovitz, C. (2015). Acupuncture during stroke rehabilitation: development of a manual for researching a complex intervention (Doctoral dissertation, University of Westminster). (Explores the relationship between complexity, EBM, and what acupuncturists do in the clinic.

**V. Other Topics**

1. Critical Thinking:

Vickers A. (1997). A proposal for teaching critical thinking to students and practitioners of complementary medicine. *Alternative therapies in health and medicine*, *3*(3), 57–62.

1. Basic Statistics

Vickers, A. (2010). *What is a P-value anyway?: 34 stories to help you actually understand statistics*. Pearson College Division.

1. Energy Medicine

Global Advances in Health and Medicine.Volume 4(Suppl); 2015 Nov (This special issue of *Global Advances in Health and Medicine* brings together many of the experts in the nascent area of biofield theory.)

Editorial

Exploring the Biofield

Mary Jo Kreitzer, Rob Saper

Glob Adv Health Med. 2015 Nov; 4(Suppl): 3–4. Published online 2015 Nov 1. doi: 10.7453/gahmj.2015.105.suppl

PMCID: PMC4654790

Introduction

Biofield Science and Healing: An Emerging Frontier in Medicine

Shamini Jain, John Ives, Wayne Jonas, Richard Hammerschlag, David Muehsam, Cassandra Vieten, Daniel Vicario, Deepak Chopra, Rauni Pritten King, Erminia Guarneri

Glob Adv Health Med. 2015 Nov; 4(Suppl): 5–7.

Original Articles

Biofield Science and Healing: History, Terminology, and Concepts

Beverly Rubik, David Muehsam, Richard Hammerschlag, Shamini Jain

Glob Adv Health Med. 2015 Nov; 4(Suppl): 8–14.

Indo-Tibetan Philosophical and Medical Systems: Perspectives on the Biofield

Shamini Jain, Jennifer Daubenmier, David Muehsam, Lopsang Rapgay, Deepak Chopra

Glob Adv Health Med. 2015 Nov; 4(Suppl): 16–24.

Biofield Science: Current Physics Perspectives

Menas C. Kafatos, Gaétan Chevalier, Deepak Chopra, John Hubacher, Subhash Kak, Neil D. Theise

Glob Adv Health Med. 2015 Nov; 4(Suppl): 25–34.

An Overview of Biofield Devices

David Muehsam, Gaétan Chevalier, Tiffany Barsotti, Blake T. Gurfein

Glob Adv Health Med. 2015 Nov; 4(Suppl): 42–51.

Clinical Studies of Biofield Therapies: Summary, Methodological Challenges, and Recommendations

Shamini Jain, Richard Hammerschlag, Paul Mills, Lorenzo Cohen, Richard Krieger, Cassandra Vieten, Susan Lutgendorf

Glob Adv Health Med. 2015 Nov; 4(Suppl): 58–66.

A Consideration of the Perspectives of Healing Practitioners on Research Into Energy Healing

Sara L. Warber, Rosalyn L. Bruyere, Ken Weintrub, Paul Dieppe

Glob Adv Health Med. 2015 Nov; 4(Suppl): 72–78.

Barriers to the Entry of Biofield Healing Into “Mainstream” Healthcare

David J. Hufford, Meredith Sprengel, John A. Ives, Wayne Jonas

Glob Adv Health Med. 2015 Nov; 4(Suppl): 79–88.

Challenges and Opportunities Faced by Biofield Practitioners in Global Health and Medicine: A White Paper

Erminia Guarneri, Rauni Prittinen King

Glob Adv Health Med. 2015 Nov; 4(Suppl): 89–96.

Review Articles

Biofield Physiology: A Framework for an Emerging Discipline

Richard Hammerschlag, Michael Levin, Rollin McCraty, Namuun Bat, John A. Ives, Susan K. Lutgendorf, James L. Oschman

Glob Adv Health Med. 2015 Nov; 4(Suppl): 35–41.

Challenges for Preclinical Investigations of Human Biofield Modalities

Gloria Gronowicz, William Bengston, Garret Yount

Glob Adv Health Med. 2015 Nov; 4(Suppl): 52–57.

Distant Healing Intention Therapies: An Overview of the Scientific Evidence

Dean Radin, Marilyn Schlitz, Christopher Baur

Glob Adv Health Med. 2015 Nov; 4(Suppl): 67–71.

Hammerschlag, R., Jain, S., Baldwin, A. L., Gronowicz, G., Lutgendorf, S. K., Oschman, J. L., & Yount, G. L. (2012). Biofield research: A roundtable discussion of scientific and methodological issues. The Journal of Alternative and Complementary Medicine, 18(12), 1081-1086.

1. Capra, F. (2010). The Tao of physics: An exploration of the parallels between modern physics and eastern mysticism. Shambhala publications.
2. Moore, G. F., Audrey, S., Barker, M., Bond, L., Bonell, C., Hardeman, W., ... & Baird, J. (2015). Process evaluation of complex interventions: Medical Research Council guidance. bmj, 350.
