# Supplementary material for: “Bringing greater research fluency into our educational vision”: A qualitative research study on improving Traditional Chinese Medicine research education
Source: PLoS One. 2024 Dec 19;19(12):e0312083. doi: 10.1371/journal.pone.0312083 (PMC11658634; doi:10.1371/journal.pone.0312083)
Supplement: S2 File — (DOCX) [file pone.0312083.s002.docx]

Original Curriculum

Major topics to cover for EAM students:*

- Why research is important for acupuncturists
- Paradoxes in Acupuncture Research based on the SAR article authored by H Langevin et al.
- History of Acupuncture Research (slide show is available)
- Physiology of acupuncture: what we have learned from basic science research on how acupuncture works:
  - Endocrine system
  - Nervous system
  - Immune system
  - Propagation of signals through tissue
- Clinical application of acupuncture: what we have learned from clinical trials on acupuncture’s effect on disease states.
  - Pain
    - Acupuncture Trialists Collaborative
    - Headache-Individual Patient Data Meta-Analysis
    - Back pain - lessons from GERAC
    - Arthritis
    - Cancer-related pain
  - Anxiety and Depression
  - Hypertension
  - Cancer Care
  - Women’s and Men’s Health
  - The NADA protocol, Battlefield Acupuncture and community acupuncture
- Whole Person Research: what we have learned from clinical trials on acupuncture’s effect on sense of well-being of patient; qualitative research on patients’ perspectives.
  - *NCCIH says whole health*
- Racial *(health)* disparities in health, health care and health access: serving communities of color
- Challenges in acupuncture research
  - Multiple active components of an acupuncture treatment; poor definition of basic terms; how points are chosen; what parts of a treatment are associated with outcome; what type of clinical research best captures which outcomes.
  - Randomized controlled trials using sham acupuncture. What are the best controls to use for acupuncture research?
  - Reframing the placebo effect.
  - Complexity Science and East Asian Medicine
- What information can we provide through acupuncture research? What do the public, medical professionals and insurance companies need to know?
  - *Translational medicine-bench to implementation science*
    - *pragmatic research-implement tx into the larger system, and the EHR*

Barriers and facilitators

- Experiential research/hands-on project for students
  - *case study following CARE and support for publication. JAIM, Medical Acupuncture*

*This presumes that students have basic research knowledge, including: basic research designs and their uses, how to select evidence, basic research statistics, understanding primary and secondary research. For detailed information on this type of course, see Basic Research Literacy folder in SAR SIG-Edu Google Drive. Also, this information does not have to be a separate course. Individual topics could be incorporated into existing courses. The target audience for this course is clinical Masters or Doctoral level acupuncture students, not Ph.D. students preparing to conduct their own research.
