# Supplementary material for: “Bringing greater research fluency into our educational vision”: A qualitative research study on improving Traditional Chinese Medicine research education
Source: PLoS One. 2024 Dec 19;19(12):e0312083. doi: 10.1371/journal.pone.0312083 (PMC11658634; doi:10.1371/journal.pone.0312083)
Supplement: S3 File — (DOCX) [file pone.0312083.s003.docx]

S3 - Characteristics of Subject Matter Experts (SMEs) and Stakeholders

**17 SME Respondents**

| Male | Female |
| --- | --- |
| 9 | 8 |

Highest Degree:

| PhD | MD | MD/PhD | DAOM |
| --- | --- | --- | --- |
| 10 | 3 | 2 | 2 |

Positions:

| Professor/Science  Researcher | Independent researcher/  acupuncturist/scholar | Medical Doctor | Research Scientist | Acupuncture practitioner |
| --- | --- | --- | --- | --- |
| 12 | 2 | 2 | 1 | 13 |

**4 Stakeholder organizations**

The following national organizations responded:

- The Accreditation Commission for Acupuncture and Herbal Medicine (ACAHM) serves as the nationally recognized accrediting agency of programs in acupuncture and East Asian Medicine (EAM) and institutions exclusively providing EAM-related programs. One board member responded to our request for comments.
- The Council of Colleges of Acupuncture and Herbal Medicine advances the standing of acupuncture and herbal medicine in the U.S. by promoting educational excellence within their membership, comprising acupuncture and herbal medicine programs in the U.S. One board member responded to our request for comments.

There are two regulatory professional organizations, ACAHM and The National Certification Commission for Acupuncture and Oriental Medicine (NCCAOM.) NCCAOM validates entry-level competency through nationally administered board exams. They are prohibited by their accrediting board, the National Commission for Certifying Agencies, from participating in the educational requirements for the acupuncture profession and therefore declined our request for comments.

In addition to these national organizations, The Society for Acupuncture Research’s Special Interest Group for Educators is a group of acupuncturists, professors, and researchers that formed at the SAR Symposium in June 2019, to improve the teaching of research skills in general and acupuncture research in particular to East Asian Medicine (EAM) students. The research team from this group conducted this current research. Two at-large members responded to our request for comments.

Conflicts of interest: as doctors, educators, and researchers, the SMEs and Stakeholders have an interest in improving the teaching of acupuncture research, and thus educating future practitioners, educators, and researchers.
