# Supplementary material for: “Bringing greater research fluency into our educational vision”: A qualitative research study on improving Traditional Chinese Medicine research education
Source: PLoS One. 2024 Dec 19;19(12):e0312083. doi: 10.1371/journal.pone.0312083 (PMC11658634; doi:10.1371/journal.pone.0312083)
Supplement: S4 File — (DOCX) [file pone.0312083.s004.docx]

Survey Instrument Details

The seeds for this survey were planted at the SAR 2019 conference. A pre-conference workshop  for acupuncture educators resulted in the formation of the SAR SIG-Edu, comprising 30  educators and administrators from approximately 18 acupuncture schools. Over four months,  members were asked to contribute to a Google document listing major topics to be included in  an acupuncture research curriculum and to recommend foundational research papers on those  topics. This document became what we will now refer to as the "Model Curriculum."

A research team of four members (HM, BA, RO, LC) refined the Model Curriculum and invited SMEs for each topic area to opine on the model curriculum. SMEs were identified by their  contributions to the field based on published articles, books, and public speaking. One of the  authors of this paper (BA) contributed as an SME. We also developed a list of stakeholders who  would have an interest in the creation of a model curriculum. This list included representatives  from EAM national organizations including the Council of Colleges of Acupuncture and Herbal  Medicine (CCAHM), the ACAHM, the National Certification Commission for Acupuncture and  Oriental Medicine (NCCAOM), the American Society of Acupuncturists (ASA), SAR and members  of the SAR SIG-Edu. Emails were sent to SMEs and Stakeholders starting on November 30,  2021, and recruitment ended on February 2, 2022. On May 8, 2022, all respondents were asked for their informed consent to use their names on a list of respondents at the end of one  or more papers, by emailing their consent to H.M. (Written consent was received from 17  respondents.)

**Table 2: Comments solicited from SMEs and stakeholders**

| Areas for SME comment | Areas for Stakeholder comment |
| --- | --- |
| Please look at the entire curriculum and  comment on it. | Please look at the entire curriculum and  comment on it. This is not necessarily a  stand-alone course, which many schools will  be unable to implement. Please view it as a  list of topics that can be presented in |

7

|  | different existing courses in a curriculum. |
| --- | --- |
| If you were designing an acupuncture  research course, what 3 papers would you  want students to read in your area of  expertise? Please write them down. | Are there topics that you would like to see  included in this list? Are there topics that you  do not think are necessary? |
| Please summarize 3-4 lessons learned in your  area of expertise. | What topics would you like to have as stand alone presentations that could be inserted in  existing courses? For example, we already  have a stand-alone presentation on the  History and Current State of Acupuncture  Research, which could be shared with  schools. |
|  | Are there any other suggestions you have for  improving acupuncture students’ knowledge  of acupuncture research? |
